# Exploratory analysis of predictors of ventricular aneurysm in a cohort of 291 patients with acute myocardial infarction

**DOI:** 10.1186/s12872-024-04002-x

**Published:** 2024-07-04

**Authors:** Hongqin Huang, Min Xu, Chaohua Qiang, Zhenni Yang, Ling Yang

**Affiliations:** 1 Department of Echocardiography and Cardiovascular Division, The Third Affiliated Hospital of Soochow University, Chang Zhou, Jiangsu 213000 China; 2 Department of Cardiovascular Division, The Third Affiliated Hospital of Soochow University, Chang Zhou, Jiangsu 213000 China

**Keywords:** Electrocardiogram, Acute myocardial infarction, Ventricular aneurysm, Total cholesterol, Predictive model

## Abstract

**Objective:**

In this study, we explored the determinants of ventricular aneurysm development following acute myocardial infarction (AMI), thereby prompting timely interventions to enhance patient prognosis.

**Methods:**

In this retrospective cohort analysis, we evaluated 297 AMI patients admitted to the First People’s Hospital of Changzhou. The study was structured as follows. Comprehensive baseline data collection included hematological evaluations, ECG, echocardiography, and coronary angiography upon admission. Within 3 months post-AMI, cardiac ultrasounds were administered to detect ventricular aneurysm development. Univariate and multivariate logistic regression analysis were employed to pinpoint the determinants of ventricular aneurysm formation. Subsequently, a predictive model was formulated for ventricular aneurysm post-AMI. Moreover, the diagnostic efficacy of this model was appraised using the ROC curves.

**Results:**

In our analysis of 291 AMI patients, spanning an age range of 32–91 years, 247 were male (84.9%). At the conclusion of a 3-month observational period, the cohort bifurcated into two subsets: 278 patients without ventricular aneurysm and 13 with evident ventricular aneurysm. Distinguishing features of the ventricular aneurysm subgroup were markedly higher values for age, B-type natriuretic peptide(BNP), Left atrium(LA), Left ventricular end-diastolic dimension (LEVDD), left ventricular end systolic diameter (LVEWD), E-wave velocity (E), Left atrial volume (LAV), E/A ratio (E/A), E/e ratio (E/e), ECG with elevated adjacent four leads(4 ST-Elevation), and anterior wall myocardial infarction(AWMI) compared to their counterparts (*p* < 0.05). Among the singular predictive factors, total cholesterol (TC) emerged as the most significant predictor for ventricular aneurysm development, exhibiting an AUC of 0.704. However, upon crafting a multifactorial model that incorporated gender, TC, an elevated ST-segment in adjacent four leads, and anterior wall infarction, its diagnostic capability: notably surpassed that of the standalone TC, yielding an AUC of 0.883 (z = -9.405, *p* = 0.000) as opposed to 0.704. Multivariate predictive model included gender, total cholesterol, ST elevation in 4 adjacent leads, anterior myocardial infarction, the multivariate predictive model showed better diagnostic efficacy than single factor index TC (AUC: 0. 883 vs. 0.704,z =-9.405, *p* = 0.000), it also improved predictive power for correctly reclassifying ventricular aneurysm occurrence in patients with AMI, NRI = 28.42% (95% CI: 6.29-50.55%; *p* = 0.012). Decision curve analysis showed that the use of combination model had a positive net benefit.

**Conclusion:**

Lipid combined with ECG model after myocardial infarction could be used to predict the formation of ventricular aneurysm and aimed to optimize and adjust treatment strategies.

**Supplementary Information:**

The online version contains supplementary material available at 10.1186/s12872-024-04002-x.

## Introduction

Acute myocardial infarction (AMI) refers to a condition where coronary artery blood supply is significantly reduced or completely interrupted due to various causes, resulting in severe and prolonged acute ischemia of the corresponding myocardium, leading to the necrosis of myocardial cells. It ranks [1, 2] as the leading cause among cardiovascular threats to human health. Early detection and treatment of complications following a myocardial infarction (MI) are critical determinants of patient outcomes.

Prompt revascularization can counteract left ventricular remodeling, improve ischemic blood flow around the infarcted area, inhibit the progression of MI, and ameliorate left ventricular morphology and contractile function [3]. However, in some MI patients, revascularization is unsuccessful, resulting in a loss of normal myocardial contractility. As time progresses, the affected area becomes supplanted by fibrous and scar tissue. Under the influences of cardiac contraction and ventricular pressure, this region becomes thinner, manifesting as cystic or irregular morphological alterations. Such aberrant motion is defined as a true ventricular aneurysm [4]. Studies have shown that the incidence of ventricular aneurysms in survivors of AMI ranges from 3.5 to 38% [5]. Research from the Mayo Clinic has revealed that patients with ST-segment elevation myocardial infarction (STEMI) have a higher incidence of ventricular aneurysm compared to non-ST-segment elevation myocardial infarction(NSTEMI) patients (OR: 1.57 vs. 1.13, *p* < 0.05). Moreover, ventricular aneurysms are more frequently observed in anterior wall myocardial infarction (AWMI) cases (31%) [6]. The 5-year survival rate for AMI patients with a ventricular aneurysm stands at 47%, while the 10-year rate is just 18% [7]. Ventricular aneurysms can lead to complications like deterioration of cardiac function, cardiac rupture, malignant arrhythmias, embolism, and death [6, 8, 9].

Therefore, early identification and prediction of ventricular aneurysm formation in hospitalized patients are vital for reducing post-MI complication rates and improving patient prognosis. In the present study, we established a predictive model for ventricular aneurysm formation after AMI by retrospectively analyzing clinical information, hematological biomarkers, and echocardiographic indicators of AMI patients upon admission. The model aimed to expedite the recognition of critical risk factors for ventricular aneurysms, thereby prompting timely interventions to enhance patient prognosis.

## Methods

### Research objects

**Fig. 1:**
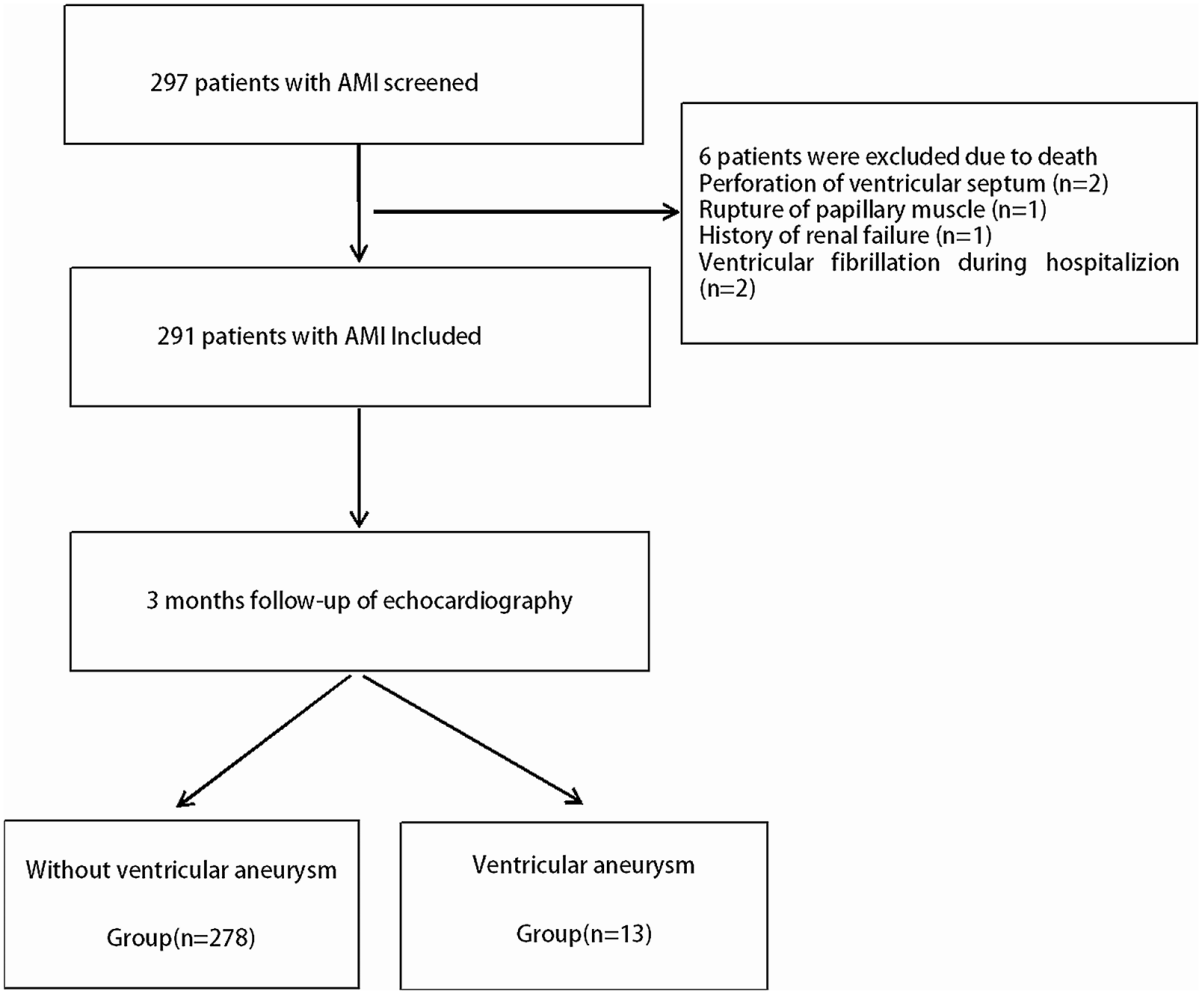
Flowchart of the study

We conducted a retrospective analysis of 297 patients, 99 ST-segment elevation myocardial infarction(STEMI) and 198 non-ST-segment elevation myocardial infarction(NSTEMI), aged 32 to 91 years, diagnosed with AMI. These patients were admitted to the Department of Cardiology at Changzhou First People’s Hospital (the Third Affiliated Hospital of Soochow University Medical College) between January and June 2020. In this cohort, 251 were males, and 46 were females. The inclusion criteria were as follows. Patients were included if they were diagnosed with AMI according to the “Diagnostic and Treatment Guidelines for ST-segment Elevation Myocardial Infarction (STEMI) 2019” [10] and the “2020 European Society of Cardiology Guidelines for the Management of Non-ST-segment Elevation Acute Coronary Syndromes” [11]. All enrolled patients underwent a thorough clinical assessment upon admission, encompassing biomarkers for myocardial injury, electrocardiogram (ECG), echocardiogram, and coronary angiogram. Exclusion criteria encompassed patients in terminal stages of malignant tumors; those with severe hematological or immunological diseases; post-cardiac surgery patients; individuals with pseudo-ventricular aneurysms, congenital left ventricular diverticula, or other severe cardiac conditions such as hypertrophic cardiomyopathy, aortic dissection, and pronounced aortic valve stenosis. Moreover, patients lacking echocardiographic data were also excluded. The procedures followed were in accordance with the Declaration of Helsinki (2008). Ethical approval was obtained from the Human Research Ethics Committee of the First People’s Hospital of Changzhou. Reference number: (2020) No.032 (quick trial). The ethics review boards in The Third Affiliated Hospital of Suzhou University / Changzhou First People’s Hospital approved their participation. Each patient provided written informed consent for long-term follow-up. This study was registered (ChiCTR2000038729.)The Date of Registration was 2020-09-29. The study flowchart is shown in Fig. 1.

## Clinical data collection

Data on patients’ medical history, laboratory test results, echocardiograms, and interventional diagnostic and therapeutic procedures were collected using the Lian Zhong Medical Record Management System of our hospital. According to the 2020 ISH Global Hypertension Practice Guidelines [12], hypertension was characterized by a systolic blood pressure (SBP) of ≥ 140 mmHg and/or a diastolic blood pressure (DBP) of ≥ 90 mmHg. To confirm this diagnosis, 2–3 clinic visits were required over a span of 1–4 weeks, contingent on the blood pressure readings. However, if the blood pressure exceeded or equaled 180/110 mmHg and there was evidence of cardiovascular disease, a diagnosis could be established in a single visit. Patients with a history of hypertension and currently under antihypertensive medication were also included in this definition. Smoking was categorized as a daily intake of ≥ one cigarette in the year prior to the current hospitalization.

### Collection and analysis of blood samples

#### Emergency blood collection

Upon immediate admission, levels of troponin, creatine kinase-MB (CK-MB), and B-type natriuretic peptide (BNP) were determined using the Beckman dxi800 Fully Automated Chemiluminescence Immunoassay Analyzer.

#### Routine blood collection

The subsequent morning after admission, in a fasting and tranquil state, 5 mL of venous blood was collected from the antecubital vein. The lipid profile was analyzed using the Beckman AU5800 Fully Automated Biochemical Analyzer. Fasting glucose levels were measured with the Johnson Vitros V5600 Fully Automated Biochemical Analyzer, while glycated hemoglobin (HbA1c) concentrations were determined via the Pu-Men HbA1c Analyzer H9. According to the 2022 ADA Diabetes Diagnosis and Treatment Standards [13], diabetes was diagnosed if the fasting blood glucose was ≥ 6.0 mmol/L, 2-hour post OGTT blood glucose was ≥ 11.1 mmol/L, random blood glucose was ≥ 11.1 mmol/L, or if the A1C level was ≥ 6.5% (48 mmol/mol). Moreover, individuals with a prior diabetes diagnosis, those on a dietary regimen, or those using oral hypoglycemic agents or insulin therapy were also recognized.

### Standard ECG

ECG assessments were conducted using the FUKUDA DENSHI (FX-8322) 12-channel electrocardiograph, employing a 12-lead configuration. The machine settings were adjusted to amplitude at 1 mV, gain at 10 mm/mV, and paper speed set to 25 mm/s. Patients were positioned supine at rest with the anterior chest region fully exposed. Prior to electrode attachment, skin sites were prepped with alcohol swabs. The 12-lead ECG electrodes were then accurately placed. The QRS wave served as the reference point for the ST segment, which was measured 80ms after the J point, in order to document any alterations in ST segment shape and elevation. These measurements spanned three cardiac cycles and were then averaged. Depending on clinical requirements, additional leads specific to the right ventricle and posterior wall were occasionally incorporated. Diagnostic criteria for ST-segment elevation included elevation of ≥ 0.2 mV in precordial leads V1 through V3 and ≥ 0.1 mV in leads V4 to V6 and limb leads, with the exception of aVR.

### Transthoracic echocardiography

After stabilization post-admission, initial echocardiographic examinations were performed using the Philips Epiq 7 C echocardiograph (the Netherlands). Patients, while resting, were placed in the left lateral decubitus position and connected to a 12-lead ECG to facilitate image capture. An X5-1 transducer was utilized, set to a depth of 16 cm and a frame rate of 50 frames/s, images spanning five cardiac cycles were obtained. These images included standard 2D and Doppler flow measurements, capturing aspects like the left ventricular longitudinal axis, aortic short axis, short axis views, five-chamber, and apical four-chamber views. Calculations were derived from these recorded metrics. At the 3-month mark following AMI, a subsequent echocardiography was performed to verify the existence of true ventricular aneurysm, which is different from pseudo-ventricular aneurysm. The diagnostic criteria for true ventricular aneurysms included: (1) thinned ventricular wall, with continuity maintained, exhibiting localized protrusion and either no movement or paradoxical motion; (2) a relatively wide neck of the aneurysm, its longitudinal diameter not shorter than the maximum diameter of the aneurysm cavity; and (3) a discernible turning point between the paradoxically moving and normally contracting walls [14]. Ventricular aneurysm was diagnosed by two senior doctors who had worked for more than five years and had intermediate title or above.

### Coronary angiography and GENSINI scoring

Coronary angiography and the evaluation of vascular stenosis were jointly undertaken by two seasoned attending physicians and technical experts. A stenosis of 50% or greater was deemed significant. The severity of coronary artery constriction was gauged using the GENSINI scoring system: a score below 20 was considered indicative of mild stenosis, a score between 20 and 50 was classified as moderate stenosis, and a score exceeding 50 was recognized as severe stenosis.

### Treatment and follow-up

Upon admission, guided by clinical indications, various reperfusion strategies were administered, such as thrombolytic therapy, percutaneous coronary intervention (PCI), or elective coronary artery bypass grafting (CABG). Supplementary therapeutic measures included antithrombotic treatments, anti-ischemic interventions, lipid modulation for plaque stabilization, enhancements to ventricular remodeling, all patients within 3 months following the MI.

### Statistical analysis

Statistical analyses were conducted using R3.4 (http://www.R-project.org/). For continuous variables with a normal distribution, results were expressed as mean ± standard deviation (SD). In cases where the distribution was non-normal, they were denoted as median (25th percentile, 75th percentile). Continuous variables across groups were compared using the unpaired Student’s t-test or the Mann-Whitney non-parametric test, as appropriate. For categorical variables, the Pearson chi-squared test or Fisher’s exact test was employed.

A multivariable logistic regression was implemented to devise a predictive model. The most fitting model parameters were chosen based on the minimum Akaike Information Criterion (AIC). To internally validate the model, bootstrap resampling (*N* = 500) was used, following the guidelines of the TRIPOD report. For quantitative variables and constructed models, ROC curves were delineated, and the diagnostic efficacy, as well as the area under the curve (AUC), was contrasted between the variables and models. List the confusion matrix to evaluate the performance of the model. Finally, decision curve analysis was performed for the prognostic model with the best discriminative abilities. Such analyses provide insight into the range of predicted risks for which the model has a higher net benefit than simply either classifying all patients as having the outcome or no (zero) patients as having the outcome. *p* -value of < 0.05 was deemed to indicate statistical significance.

## Results

In this study, a total of 291 cases of AMI patients were included, comprising 247 males and 44 females. Two patients had ventricular septal perforation, 1 patient had papillary muscle rupture, 1 patient had a history of renal failure, and 2 patients had ventricular fibrillation after admission. The above patients were not included in the study. Based on the 3-month follow-up results from echocardiography, patients were categorized into two groups: the non-ventricular aneurysm group (278 cases) and the ventricular aneurysm group (13 cases). A comparison of clinical data, hematological parameters, and echocardiographic parameters between the two groups is presented in Table 1. The ventricular aneurysm group exhibited statistically significant differences (*p* < 0.05) compared to the non-ventricular aneurysm group in terms of age, BNP levels, left atrial size, left ventricular end-diastolic diameter (LVEDD), E-wave velocity, left atrial volume (LAV), E/A ratio, E/e ratio, and 4 ST-segment elevation (4 ST Elevation) in four consecutive leads on the ECG, all of which were higher in the ventricular aneurysm group. Conversely, the ventricular aneurysm group showed lower levels of total cholesterol (TC), ejection fraction (EF), interventricular septum thickness, and the proportion of inferior wall myocardial infarction (IWMI) compared to the non-ventricular aneurysm group, with statistically significant differences (*p* < 0.05).

**Table 1:** Comparison of general information of AMI patients

Variables	NO Ventricular aneurysm Group	Ventricular aneurysm Group	*p*-value
N	278	13	
Age(years)	60.849 ± 12.294	67.692 ± 11.183	0.051
BMI (kg/m2)	25.081 ± 4.114	23.808 ± 3.264	0.172
Male, n (%)	234 (84.173%)	13 (100.000%)	0.119
Smoke, n (%)	156 (56.115%)	7 (53.846%)	0.872
Drinking history, n (%)	27 (9.747%)	2 (15.385%)	0.508
Hypertension, n (%)	223 (80.216%)	10 (76.923%)	0.771
Diabetes mellitus, n (%)	132 (47.482%)	7 (53.846%)	0.653
SBP(mmHg)	130.935 ± 24.208	126.917 ± 19.000	0.571
DBP(mmHg)	78.986 ± 15.295	78.538 ± 10.959	0.917
CTnI(µg/L)	59.956 ± 659.073	33.097 ± 85.133	0.884
CK-MB(U/L)	16.000 (1.600-109.250)	2.500 (1.300–11.900)	0.024
FBG (mmol/L)	6.642 ± 3.415	8.098 ± 3.105	0.133
TC (mmol/L)	3.960 ± 1.354	3.202 ± 1.140	0.048
Triglycerides (mmol/L)	1.495 (1.040–2.045)	1.210 (1.030–1.670)	0.338
HDL (mmol/L)	0.974 ± 0.402	0.893 ± 0.438	0.481
VLDL (mmol/L)	2.325 (1.643–2.938)	1.900 (1.630–2.270)	0.262
Apo-A (mmol/L)	0.958 ± 0.361	0.818 ± 0.328	0.175
Apo-B (mmol/L)	0.811 ± 0.333	0.686 ± 0.298	0.187
BNP (pg/ml)	127.000 (0.000-479.000)	476.000 (0.000-1395.000)	< 0.001
LA(mm)	37.519 ± 4.700	40.692 ± 7.825	0.023
LVEDD(mm)	51.169 ± 5.169	55.538 ± 7.731	0.004
EF(%)	55.031 ± 8.226	39.692 ± 11.905	< 0.001
E(cm/s)	76.177 ± 20.133	104.500 ± 14.012	0.006
A(cm/s)	77.140 ± 20.137	61.500 ± 11.790	0.126
IVS (mm)	9.504 ± 1.563	8.077 ± 2.532	0.002
LAV(ml)	54.508 ± 19.923	83.500 ± 17.483	0.005
E/A	1.061 ± 0.417	1.796 ± 0.678	< 0.001
E/e	5.585 ± 1.787	7.606 ± 2.527	0.030
STEMI, n (%)	91 (32.734%)	8 (61.538%)	0.032
AWMI, n (%)	34 (12.230%)	6 (46.154%)	< 0.001
IWMI, n (%)	57 (20.504%)	2 (15.385%)	0.047
4ST-Elevation, n (%)	71 (25.540%)	10 (76.923%)	< 0.001
Gensini Score	65.451 ± 45.419	90.308 ± 45.672	0.055
Number of diseased vessels			0.179
0	14 (5.036%)	0 (0.000%)	
1	63 (22.662%)	0 (0.000%)	
2	80 (28.777%)	5 (38.462%)	
3	121 (43.525%)	8 (61.538%)	
Stent, n	1.209 ± 0.950	1.231 ± 1.013	0.935

Data were presented as mean ± SD or P50(P25, P75)or n(%)

BMI, Body mass index; SBP, Systolic blood pressure; DBP, Diastolic blood pressure; CTn-I, cardiac troponin I; CK-MB, Creatine kinase-MB; FBG, Fasting blood glucose; HDL, High density lipoprotein-cholesterol; TC, total cholesterol; VLDL, Very low density lipoprotein; Apo A, Apolipoproteins A; ApoB, Apolipoproteins B; BNP, B-type natriuretic peptide; LA, Left atrium; LVEDD, Left ventricular end-diastolic dimension; LVEWD, left ventricular end systolic diameter; EF, Ejection fraction; E,E-wave velocity; A, A-wave velocity; IVS, interventricular septum; LAV, Left atrial volume; E/A, E/A ratio; E/e, E/e ratio; STEM, ST segment elevation myocardial infarction; AWMI, anterior wall myocardial infarction; IWMI, inferior wall myocardial infarction;4 ST-Elevation, ECG with elevated adjacent four leads

### Multivariable logistic regression for predicting ventricular aneurysm formation in AMI patients

Bivariate multivariate Logistic analysis showed that TC, ST-segment elevation in 4 leads and anterior myocardial infarction in AMI patients were independent indicators of ventricular aneurysm formation (*p* ≤ 0.05)(Table 2).

**Table 2:** Multivariate Logistic regression analysis of influencing factors of ventricular aneurysm formation after acute myocardial infarction

Variables	Regression coefficient	Sem	OR	OR(95%CI)	*p* value
AWMI	2.399	1.111	10.989	1.247–96.873	0.031
4ST-Elevation	2.850	1.090	17.344	2.049-146.803	0.009
TC	-0.542	0.244	0.578	0.359–0.932	0.025
STEMI	1.654	0.981	5.284	0.773–36.128	0.090

AWMI, anterior wall myocardial infarction; 4 ST-Elevation, ECG with elevated adjacent four leads; TC, total cholesterol; STEM, ST segment elevation myocardial infarction

**Fig. 2:**
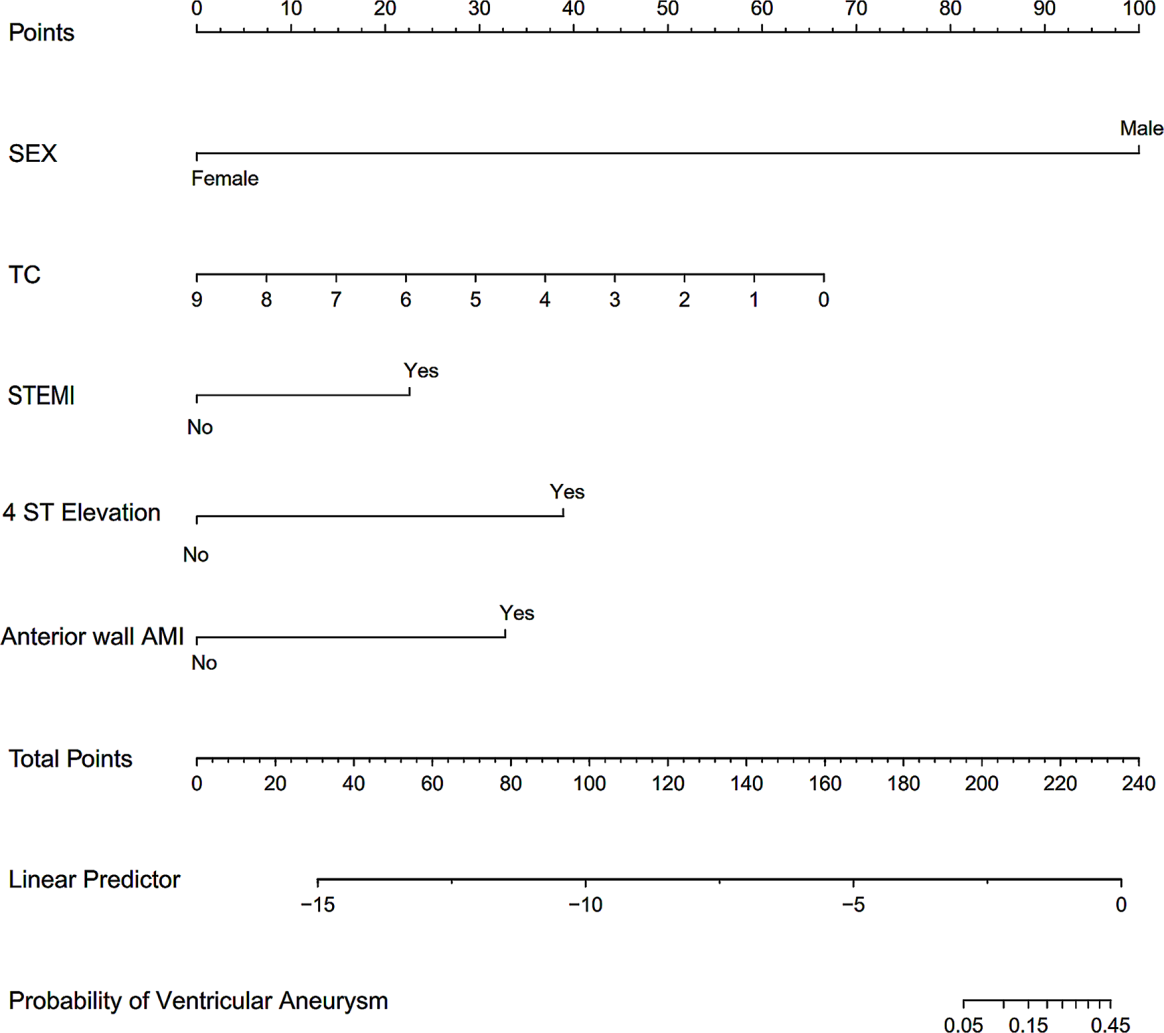
The nomogram of the prediction model for ventricular aneurysm formation after AMI.A score is given to each value level for each predictor on the left; For multiple predictors of each patient, a total score can be calculated, and the probability of developing a ventricular aneurysm in each patient with AMI can be calculated from the score

A predictive model was constructed using five variables: gender, TC, STEMI, the presence of ST-segment elevation in four leads, and AWMI. A stepwise (stepAIC) selected model was formulated as follows: Logit(P) = -5.052–16.161×(Female = 1) − 0.542×TC + 1.654× (STEMI = 1) + 2.850 × (4ST-Elevation = 1) + 2.399×(AWMI = 1). The nomogram of the predictive model for ventricular aneurysm formation in AMI patients is illustrated in Fig. 2. The Confusion Matrix of the prediction model is shown in Table S1.

### ROC curve comparison and net reclassification improvement (NRI) analysis

**Fig. 3:**
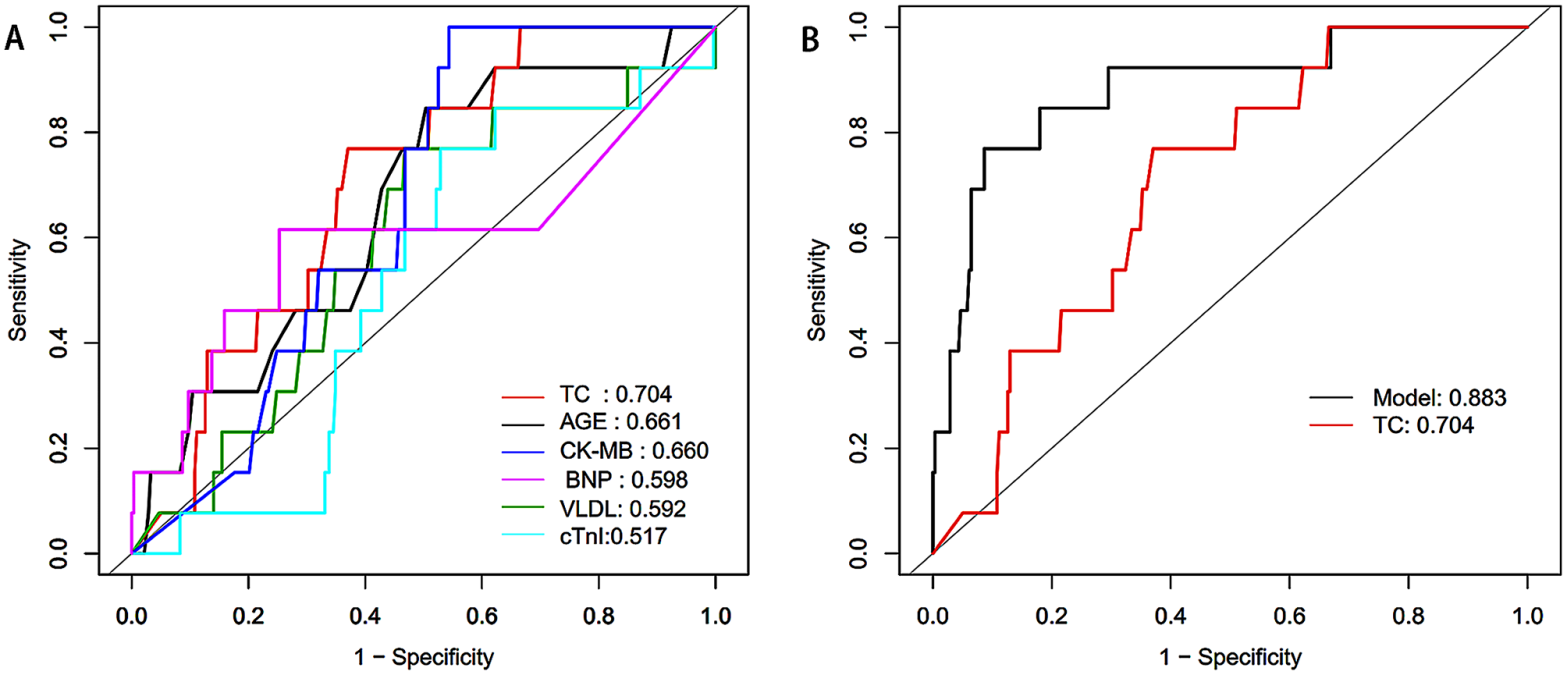
Comparison of the ROC curves of single factors (**A**) and combination model (**B**) in predicting ventricular aneurysm after AMI. (**A**) ROC curve analysis was performed for 6 single factors TC, AGE, CK-MB, BNP, VLDL and troponin; (**B**) ROC curve analysis of optimal single univariate index TC and combination prediction model

The study began with an ROC curve analysis of six quantitative indices in AMI patients: TC, age, CK-MB, BNP, VLDL, and CTn-I. The analysis revealed that TC had the highest AUC value. Following this, we compared the diagnostic performance of TC, the optimal single factor, with that of a combined predictive model using ROC curve analysis. This comparison demonstrated that the combined predictive model outperformed TC alone, showing an AUC of 0.883 versus 0.703. This difference was statistically significant (z = -9.405, *p* = 0.000) (Table 3; Fig. 3). Then, we compared univariate TC with multivariate model containing sex, TC, adjacent 4-lead ST elevation, STEMI, and anterior myocardial infarction. Multivariate model improved continuous net reclassification index Net reclassification improvement (NRI) is 28.42% (95% CI: 6.29-50.55%; *p* = 0.012), which correctly reclassifying aneurysm incidence improved prediction by 29.56% (*p* = 0.017), while correctly reclassifying aneurysm absence improved prediction by-1.14% (*p* = 0.052).

**Table 3:** ROC curve to evaluate the efficacy of univariate in predicting true ventricular aneurysm after AMI

Test	AUC	95%CI	Best threshold	Specificity	Sensitivity	Accuracy
TC (mmol/L)	0.704	0.587–0.821	3.68	0.630	0.769	0.636
Age	0.661	0.517–0.804	59.5	0.496	0.846	0.512
CK-MB(U/L)	0.660	0.561–0.758	25.45	0.457	1.000	0.481
BNP (pg/ml)	0.598	0.393–0.803	470	0.747	0.615	0.741
VLDL (mmol/L)	0.592	0.437–0.748	2.275	0.532	0.769	0.543
CTnI(µg/L)	0.517	0.380–0.654	8.49	0.471	0.769	0.485

TC, total cholesterol; CK-MB, Creatine kinase-MB; BNP, B-type natriuretic peptide; VLDL, Very low density lipoprotein; CTn-I, cardiac troponin I;

### Decision curve (DCA) and calibration curve analysis

**Fig. 4:**
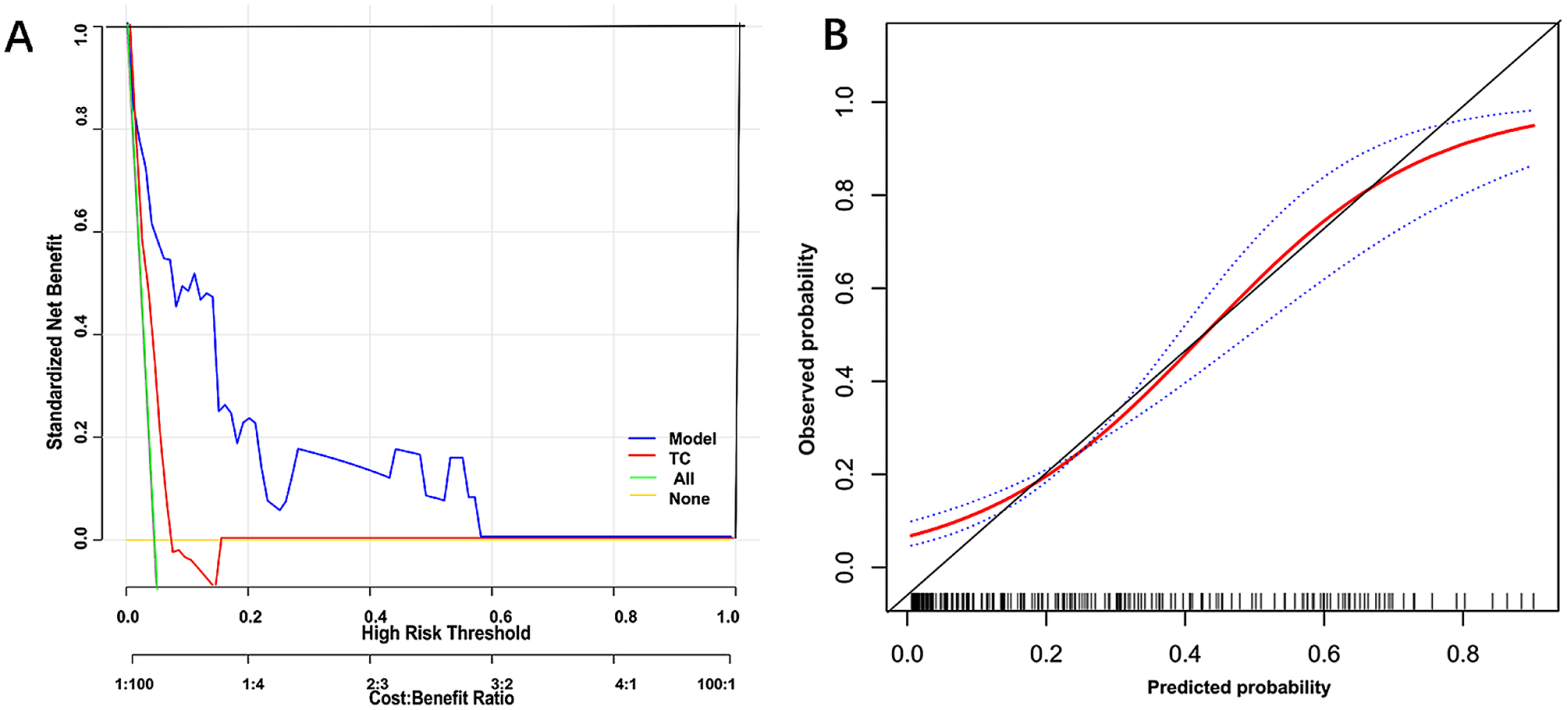
Decision curve analysis of the prognostic model of ventricular aneurysm in patients with AMI. For the single factor, combination prediction model shows the net return curve. Solid brown line = net benefit when all AMI patients consider no outcome (ventricular aneurysm); Solid green line = net gain when all patients with AMI are considered to have a ventricular aneurysm. The preferred model is the one with the better net benefit at any given threshold. The calibration curve of the prediction model for identifying patients with ventricular aneurysm after AMI. *Note* The horizontal axis is the predicted incidence of AMI in patients with ventricular aneurysm, and the vertical axis is the actual incidence. The black line is the reference line, indicating that the predicted value is same as the actual value; the red line is the calibration curve, and blue dotted line is the 95% CI of the predicted value

The decision curve (DCA) confirmed that the combined model adequately identified patients with ventricular aneurysm after AMI, for predicted probability thresholds between 0% and 60%, prognostic model showed a positive net benefit. (Fig. 4A). The calibration curve illustrates good agreement between predicted and observed values of the predictive model (Fig. 4B).

## Discussion

The incidence of AMI is notably higher in males. International research indicates that the mean age of AMI patients typically falls between 60 and 69 years [15, 16]. In contrast, local studies from our region pinpoint the average age at 60.2 ± 13.7 years [17, 18]. A ventricular aneurysm is a frequent sequel of AMI. Notably, advancements in therapeutic strategies have influenced its prevalence. The European Society of Cardiology and the American College of Cardiology reports that with the broader adoption of thrombolytic therapy, PCI techniques, and enhanced medical management, the incidence of ventricular aneurysms has declined to around 5% [19, 20]. In our investigation, the incidence was slightly lower, at 4.5%. Intriguingly, about half of these aneurysms presented within the first 48 h post-MI, while the remainder manifested approximately 2 weeks post-infarction [21].

The core contribution of our research was the development of a predictive model that gauged the likelihood of ventricular aneurysm formation in AMI patients. This model amalgamated several key variables: patient gender, TC levels, STEMI, AWMI, and ST-segment elevation across four consecutive leads in the ECG. Evidence from our study strongly suggested that this model was highly efficacious in forecasting ventricular aneurysms post-AMI. For individuals identified as high risk for such aneurysms after AMI, swift and rigorous revascularization, combined with prompt intervention and treatment, could potentially decrease the incidence of aneurysm formation. By adopting these proactive measures, clinicians can also reduce the risk of severe complications, including cardiac rupture, malignant arrhythmias, embolism, and cardiogenic shock [6, 8, 9]. A salient observation is the pivotal role of AWMI in aneurysm development. The left anterior descending coronary artery, which nourishes a significant portion of the myocardium, can, upon near-midshaft subtotal or complete occlusion, induce widespread myocardial cell necrosis. This necrosis often evolves into scar tissue, acting as a precursor for ventricular aneurysm formation [22].

Zhu Tiangang et al. [23], through their research utilizing echocardiographic imaging, have delineated an independent link between disease of the anterior descending coronary artery and myocardial perfusion irregularities post-PCI. Furthermore, a study spearheaded by Sun et al. [24], which employs coronary angiography and echocardiography within the initial week of AMI patient admission, has highlighted anterior wall STEMI, disease of the anterior descending coronary artery, and double or triple vessel disease as distinct factors. Notably, the anterior wall and the culprit vessel emerge as significant risk determinants. The role of reperfusion therapy is paramount, as it not only diminishes the magnitude of myocardial necrosis but also acts as a cardinal influencer in the evolution of ventricular aneurysms.

ECG holds a central role in the diagnosis and management of MI, offering substantial clinical insights. Changes in the ST-T segments of ECG leads, corresponding to the ischemic myocardial region, effectively gauge myocardial reperfusion and act as a sensitive marker for ventricular aneurysms post-MI. Within the scope of AMI, ST-segment elevation across roughly four contiguous leads often indicates a more extensive myocardial involvement and stands as a strong predictor for the onset of ventricular aneurysms [25]. Research by A. W. van ‘t Hof et al. has established that effective reperfusion therapy, marked by ST-segment normalization, curtails myocardial damage, culminating in favorable long-term outcomes during subsequent follow-ups [25]. In tandem, work by Birnbaum Y and associates [26] has underscored the utility of the initial ECG pattern and the count of leads with ST-segment elevation in prognosticating the eventual infarct size. Our investigation aligned with these insights, emphasizing that ST-segment elevation spanning four consecutive leads signified a heightened risk for ventricular aneurysm development. Given this, it’s imperative to aggressively champion comprehensive interventions, like timely revascularization, for such patients. This cohort, given the broader myocardial damage, faced an increased likelihood of left ventricular dysfunction and augmented mortality risk during extended follow-ups.

Dyslipidemia is a primary risk factor for atherosclerosis [27]. As serum cholesterol levels rise, the severity of coronary lesions intensifies, likely due to the lipid deposits within the coronary vessel endothelium, which amplify vascular damage [28, 29]. In relation to AMI, as early as 1971, Fyfe et al. noted declines in TC during the hospitalization of patients with acute coronary syndrome [30]. Succeeding research has consistently reported decreases in TC and low-density lipoprotein cholesterol (LDL-C) levels, typically within 24 to 48 h post-AMI [31, 32].

Our research also identified a reduction in TC and LDL-C levels by the second day of hospitalization post-AMI. Such trends could be ascribed to a myriad of factors linked with the aftermath of MI. These factors encompassed adrenergically mediated lipolysis in adipose tissue, the administration of lipid-lowering medications, decreased intake of saturated fats, and shifts in lifestyle behaviors. Expanding on this, Michael Miller and associates have posited that these lipid and lipoprotein changes hinge more on the magnitude of myocardial necrosis and baseline serum lipid concentrations rather than being directly impacted by thrombolytic treatments or PCI [32].

AWMI, multi-lead ST-segment elevation, and lipid irregularities post-MI are all pivotal risk factors for ventricular aneurysm development. Yet, studies focusing on individual factors have demonstrated only modest predictive power. This implies that amalgamating these elements with other indicators can bolster the prediction of aneurysm onset. In our research, we harnessed logistic regression to devise a new predictive model for ventricular aneurysm formation. Subsequent ROC curve analysis validated that our model surpassed the predictive capacity of single-factor metrics, thus heightening both diagnostic specificity and precision.

Pujiao Yu and colleagues [33], drawing from coronary angiography outcomes, have formulated a predictive model for ventricular aneurysms. They underscore the significance of the distance of the culprit lesion to the distal coronary artery as a robust predictive factor. In a subsequent study involving AMI patients who undergo PCI, Masayuki Mor et al. [34] have found that peak CK levels, heart rate, and final TIMI flow grading are all independently linked to the onset of left ventricular apical aneurysms (LVAA). Moreover, Yuanming Xing and team [35] have proposed a ventricular aneurysm predictive model rooted in seven key parameters: age, cardiovascular disease history, left ventricular ejection fraction, ST-segment elevation, prior PCI experiences, mean platelet volume, and aspartate transaminase levels. Impressively, their model showcases robust predictive efficacy, boasting an AUC of 0.908. While the aforementioned models frequently hinge on coronary angiography, a procedure requiring significant physician expertise and numerous manual measurements, our study took a different tack. Although we initially incorporated coronary angiography results and revascularization decisions, our final model was anchored mainly in electrocardiography and post-admission lipid indicators. This methodology presented a quicker and more universally applicable strategy.

Echocardiography stands as the primary non-invasive diagnostic tool for AMI patients, playing an essential role in evaluating mechanical complications, hemodynamics, and overall cardiac function [36–39]. Moreover, myocardial contrast echocardiography (MCE) employs contrast agents to illuminate infarcted areas, further facilitating the detection of ventricular aneurysms. Positron emission tomography (PET) is adept at differentiating between hibernating and stunned myocardium while also evaluating ventricular aneurysm formation. Cardiac magnetic resonance imaging (CMR) provides detailed insights into infarct size, microvascular obstruction, intramyocardial hemorrhage, and myocardial strain metrics. Nevertheless, the widespread clinical adoption of these advanced imaging modalities remains limited. This is largely due to prolonged examination durations, radiation concerns, elevated costs, constraints in testing patients with heart failure in a supine stance, and difficulties encountered during severe arrhythmias. As a result, echocardiography continues to be the preferred diagnostic modality in regular clinical settings. Its cost-effectiveness, user-friendliness, high repeatability, and bedside suitability render it both widely accepted and accessible for assessing AMI patients.

## Conclusion

Therefore, the lipid combined with ECG model after myocardial infarction could be used to predict the formation of ventricular aneurysm, improving the accuracy and specificity of diagnosis. This model may provide some help for early identification of high-risk patients, active intervention of controllable risk factors, early prediction of ventricular aneurysm formation, and thus reduce the serious illness rate and mortality rate of patients.

There are several limitations in the present work. (1) The scope of our study’s follow-up was confined to a 3-month period, excluding evaluations of aneurysm developments beyond this duration. (2) We did not have a validation set, while we utilized the Bootstrap method for internal validation. (3)The imbalance between the non-ventricular aneurysm group (278 cases) and the ventricular aneurysm group (13 cases) may raise concerns about the potential impact on the statistical analyses.

### Electronic supplementary material

Below is the link to the electronic supplementary material.

Supplementary Material 1

## Data Availability

The data analyzed in this study are not publicly available due to the privacy policy of the hospital but are available from the corresponding author on reasonable request.

## Abbreviations

ECG: Electrocardiogram
BNP: B-type natriuretic peptide
LA: Left atrium
LVEDD: Left ventricular end-diastolic dimension
LVEWD: Left ventricular end systolic diameter
EF: Ejection fraction
E: E-wave velocity
A: A-wave velocity
IVS: Interventricular septum
LAV: Left atrial volume
E/A: E/A ratio
E/e: E/e ratio
STEMI: ST segment elevation myocardial infarction
AWMI: Anterior wall myocardial infarction
TC: Total cholesterol
NSTEMI: Non-ST-segment elevation myocardial infarction
BMI: Body mass index
SBP: Systolic blood pressure
DBP: Diastolic blood pressure
CTn-I: Cardiac troponin I
CK-MB: Creatine kinase-MB
HbA1c: Glycated hemoglobin
PCI: Percutaneous coronary intervention
CABG: Elective coronary artery bypass grafting
SD: Standard deviation
FBG: Fasting blood glucose
HDL: High density lipoprotein-cholesterol
VLDL: Very low density lipoprotein
ApoA: Apolipoproteins A
ApoB: Apolipoproteins B
IWMI: Inferior wall myocardial infarction
4 ST-Elevation: 5 ECG with elevated adjacent four leads
AIC: Akaike Information Criterion
NRI: Net reclassification improvement
DCA: Decision curve
LVAA: Left ventricular apical aneurysms
MCE: Myocardial contrast echocardiography
PET: Positron emission tomography
CMR: Cardiac magnetic resonance imaging
