# Supplementary material for: Exploratory analysis of predictors of ventricular aneurysm in a cohort of 291 patients with acute myocardial infarction
Source: BMC Cardiovasc Disord. 2024 Jul 4;24:336. doi: 10.1186/s12872-024-04002-x (PMC11223387; doi:10.1186/s12872-024-04002-x)
Supplement: Supplementary file 1 — Supplementary Material 1 [file 12872_2024_4002_MOESM1_ESM.docx]

**Supplementary Information**

**Table S1 Confusion Matrix of the prediction model to predict ventricular aneurysms in AMI patients**

|  | | True Category | |
| --- | --- | --- | --- |
|  |  | Positive(N) | Negative(N) |
| Prediction Category | Positive(N) | **10** | **3** |
|  | Negative(N) | **10** | **254** |
